# Sex differences in tau and synaptic pathologies in a humanized MAPT mouse model revealed by PET imaging

**DOI:** 10.1002/alz70862_110229

**Published:** 2025-12-23

**Authors:** Christopher D Morrone, Junchao Tong, Darcy Wear, Anton Lindberg, Sahana Kunanayagam, Mohammad Alijaniaram, Wai Haung Yu, Neil Vasdev

**Affiliations:** ^1^ Centre for Addiction and Mental Health, Toronto, ON Canada; ^2^ University of Toronto, Toronto, ON Canada

## Abstract

**Background:**

Tauopathy is a prominent feature of dementia and is present in over 20 disorders including Alzheimer’s disease (AD) and frontotemporal dementia (FTD), involving the hyperphosphorylation and aggregation of microtubule associated protein tau (MAPT). A humanized knock‐in tauopathy mouse model with FTD‐related mutations, *MAPT^10+3/S305N^* (S305N), presents with toxic 4‐repeat‐(4R) tau isoforms, phosphorylated‐tau inclusions, and synaptic degeneration. Our objective is to conduct PET imaging of female and male S305N mice for tau and synaptic pathologies, with [^18^F]OXD‐2314 and [^18^F]SynVesT‐1, respectively, and relate these findings to the behavioral and pathological phenotype to understand tauopathy progression, and potential sex differences.

**Method:**

S305N mice were compared to non‐mutated *MAPT* knock‐in mice. Mice underwent PET/CT imaging with [^18^F]OXD‐2314, advantageous to image mixed 3R/4R tau isoforms and non‐AD tau species, and [^18^F]SynVesT‐1 for synaptic vesicle glycoprotein 2A. Brain region time activity curves (TACs) were extracted in standardized uptake values (SUV). Cognition was assessed in the Barnes maze, and sleep activity patterns by home‐cage video recording. Mouse brains were collected for immunohistochemical analysis of phosphorylated‐tau (PHF1, CP13).

**Result:**

[^18^F]OXD‐2314 SUVR_30‐60min_ (cerebellum as reference region) was significantly increased in the whole brain of female S305N mice, compared to female MAPT, notably in the frontal cortex with a trend in the hippocampus, and unchanged in the entorhinal cortex. No differences in [^18^F]OXD‐2314 uptake were detected between males. [^18^F]SynVesT‐1 demonstrated significantly reduced whole brain SUV_0‐90min_ and volume of distribution in female S305N mice compared to MAPT indicative of synaptic loss, but no changes were seen in males. Cognition and sleep were impacted in both sexes of S305N mice; yet cognitive deficits were more advanced in male S305Ns. Immunohistochemistry indicated increased tau aggregates in S305N mice in the frontal cortex, hippocampus and entorhinal cortex, with no sex differences.

**Conclusion:**

PET imaging revealed more rapid tau and synaptic pathologies in female S305Ns despite greater cognitive deficits in male S305N mice. Pathological assessment for neurodegeneration is ongoing. Further characterization of [^18^F]OXD‐2314 and [^18^F]SynVesT‐1 in tauopathy mouse lines will support our PET imaging studies with these radiopharmaceuticals in patients with tauopathy.

